# Gradient-guided boundary-aware selective scanning with multi-scale context aggregation for plant lesion segmentation

**DOI:** 10.3389/fpls.2025.1727075

**Published:** 2025-12-23

**Authors:** Guanqun Sun, Tianshuo Li, Yizhi Pan, Zidan Zhu, Tianhua Yang, Feihe Shao, Jia Guo, Junyi Xin

**Affiliations:** 1 School of Information Engineering, Hangzhou Medical College, Hangzhou, Zhejiang, China; 2 School of Information Science, Japan Advanced Institute of Science and Technology, Nomi, Japan; 3 Faculty of Computer and Information Sciences, Hosei University, Tokyo, Japan

**Keywords:** gradient-guided, Mamba, multi-scale context aggregation, plant lesion segmentation, selective scanning, state space models

## Abstract

**Introduction:**

Plant lesion segmentation aims to delineate disease regions at the pixel level to support early diagnosis, severity assessment, and targeted intervention in precision agriculture. However, the task remains challenging due to large variations in lesion scale—ranging from minute incipient spots to coalesced regions—and ambiguous, low-contrast boundaries that blend into healthy tissue.

**Methods:**

We present GARDEN, a Gradient-guided boundary-Aware Region-Driven Edge-refiNement network that unifies multi-scale context modeling with selective long-range boundary refinement. Our approach integrates a Multi-Scale Context Aggregation (MSCA) module to harvest contextual cues across diverse receptive fields, forming scale-consistent lesion priors to improve sensitivity to tiny lesions. Additionally, we introduce a Boundary-aware Selective Scanning (BASS) module conditioned on a Gradient-Guided Boundary Predictor (GGBP). This module produces an explicit boundary prior to steer a Mamba-based 2D selective scan, allocating long-range reasoning to boundary-uncertain pixels while relying on local evidence in confident interiors.

**Results:**

Validated across two public plant disease datasets, GARDEN achieves state-of-the-art results on both overlap and boundary metrics. Specifically, the model demonstrates pronounced gains on small lesions and boundary-ambiguous cases. Qualitative results further show sharper contours and reduced spurious responses to illumination and viewpoint changes compared to existing methods.

**Discussion:**

By coupling scale robustness with boundary precision in a single architecture, GARDEN delivers accurate and reliable plant lesion segmentation. This method effectively addresses key challenges in the field, offering a robust solution for automated disease analysis under challenging real-world conditions.

## 1 Introduction

The goal of plant lesion segmentation is to automatically label disease regions in plant images at the pixel level. This task is important for precision agriculture: it supports early diagnosis, severity measurement, and targeted treatment, which help protect yield and reduce broad-spectrum chemicals (Khan et al., 2023; Saleem et al., 2023). However, this task is challening. On the one hand, it is a hard pattern analysis problem. The model must know what the symptoms are and where they appear. Lesions vary widely in size and look, from tiny spots to large merged areas. This is harder than image-level classification and coarse detection. On the other hand, lesion boundaries are often soft and blend into healthy tissue. Useful cues include small changes in color, texture, and shape, while viewpoint or lighting changes are mostly noise. Effective plant lesion segmentation still needs two abilities at the same time. It must gather context across multiple scales, and it must refine uncertain boundaries with help from long-range cues.

Classic U-Net and many variants remain strong baselines for pixel-level segmentation (Ronneberger et al., 2015). For plant disease images, prior work often adds multi-scale fusion or pyramid-style pooling to handle large changes in lesion size (Wang et al., 2023; Chen et al., 2023). Attention modules are also used to highlight lesion cues and suppress background noise (Wang et al., 2024). These designs improve recall of tiny spots and large regions, yet the predicted edges can still look soft. A second line models long-range context with Transformers (Cao et al., 2022) or state space models such as Mamba (Gu and Dao, 2024). Transformers capture global relations but are costly at high resolution; Mamba offers long context with lower cost, but most uses do not guide it with an explicit boundary map.

**Figure 1:**
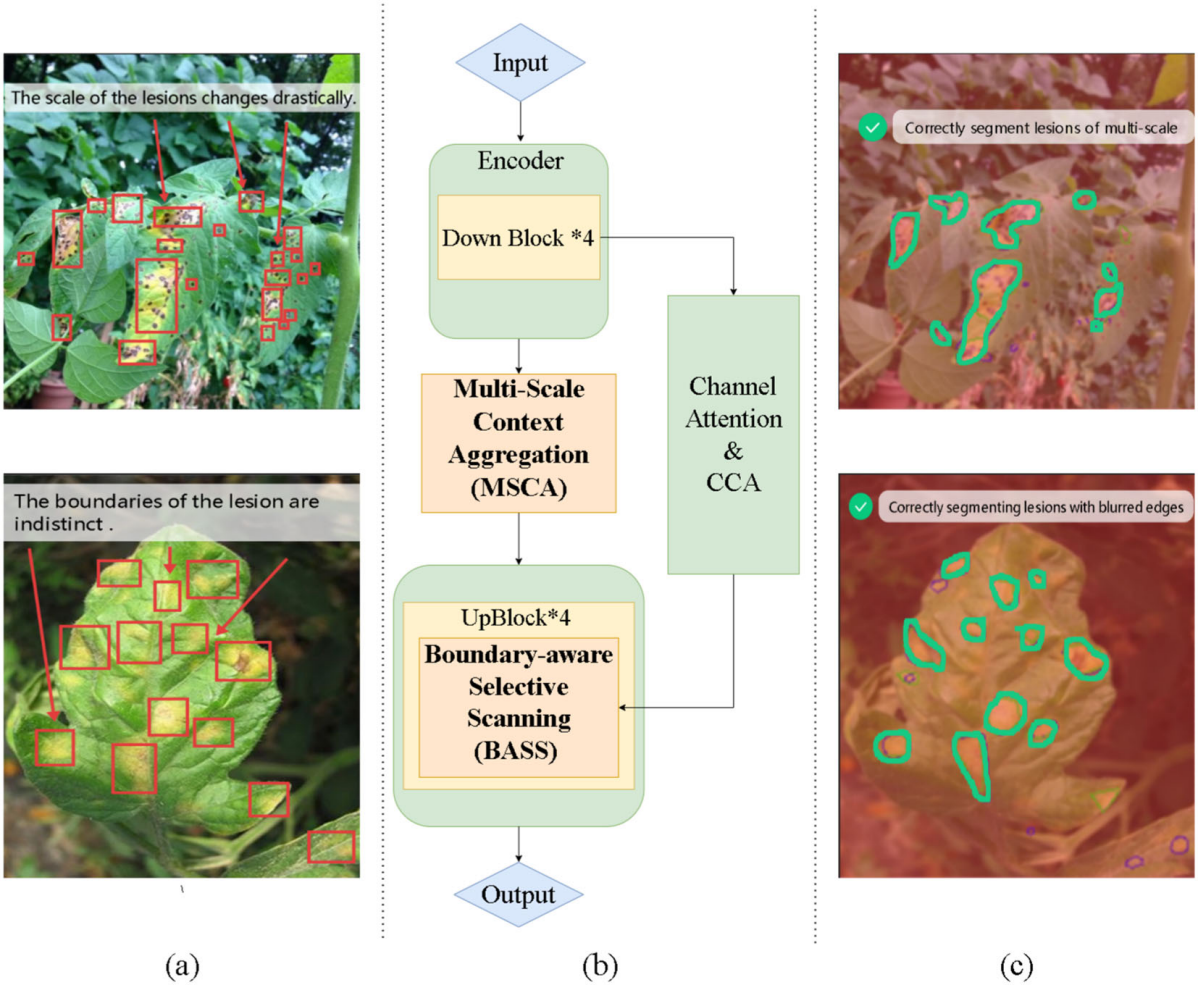
**(A)** Two core challenges in plant lesion segmentation: drastic variation in lesion scale and ambiguous, low-contrast boundaries. **(B)** Our proposed GARDEN architecture, which integrates a MultiScale Context Aggregation (MSCA) module to form scale-consistent lesion priors and a Boundary-aware Selective Scanning (BASS) module conditioned on a Gradient-Guided Boundary Predictor (GGBP) for explicit contour refinement. **(C)** Qualitative results showing that GARDEN yields precise masks across extreme scales and sharpened boundaries in challenging cases.

Despite their promising performance, there are two key limitations regarding the learning of plant lesion segmentation feature. First, the lesions exhibit drastic variations in scale, ranging from minute, incipient spots indicative of early infection to large, coalesced necrotic regions in advanced stages (**Figure 1A**). Networks with fixed receptive fields often struggle to capture this diversity, leading to either missed detections of small targets or incomplete segmentation of large ones (Chen et al., 2023). Second, lesions are often characterized by irregular shapes and ambiguous boundaries, gradually transitioning into healthy tissue without sharp edges (**Figure 1A**). This ambiguity poses a significant challenge for precise localization, which is crucial for accurate morphological analysis.

To address these two limitations, we have some insights. First, the model should harvest multi-scale context to construct scale-consistent lesion priors. Such priors expose minute, incipient spots while preserving the integrity of large, coalesced regions, enabling the network to extract discriminative features despite drastic variations in lesion size. Second, long-range reasoning ought to be applied selectively to boundary-uncertain pixels, while confident interiors rely on local evidence; this targeted allocation sharpens contours and suppresses spurious responses to illumination or viewpoint changes. Third, scale robustness and boundary precision should be combined and act jointly to improve segmentation: multi-scale context guides boundary localization, while boundary cues stabilize cross-scale aggregation.

To address the aforementioned two limitations, we propose GARDEN, a Gradient-primed boundary-Aware Region-Driven Edge refiNement network with selective scanning and multi-scale context aggregation for precise plant lesion segmentation. At its core, a Multi-Scale Context Aggregation (MSCA) (**Figure 1b**) module aggregates contextual cues across diverse receptive fields to form scale-consistent lesion priors, enabling reliable detection from minute early spots to large coalesced regions. Complementing this, a Boundary-aware Selective Scanning (BASS) (**Figure 1b**) module employs a Gradient-Guided Boundary Predictor (GGBP) to produce an explicit boundary prior and uses it to condition a Mamba-based 2D selective scan—allocating long-range modeling capacity to boundary-uncertain pixels while letting confident interiors rely on local evidence. This unified design couples multi-scale reasoning with boundary-guided long-range refinement, yielding crisp contours and accurate masks across extreme scale variation (**Figure 1c**).

The main contributions of this work are summarized as follows:

- We propose GARDEN, a Gradient-Guided boundary-aware Region-Driven Edge-refinement network with selective scanning and multi-scale context aggregation—a unified model that jointly addresses the two core challenges of plant lesion segmentation: drastic lesion-scale variation and ambiguous, low-contrast boundaries.
- We design a Multi-Scale Context Aggregation (MSCA) module to harvest cross-scale context and form scale-consistent lesion priors, and a Boundary-aware Selective Scanning (BASS) module conditioned on a Gradient-Guided Boundary Predictor (GGBP) to allocate long-range reasoning selectively to boundary-uncertain pixels for explicit contour refinement.
- We conduct extensive experiments on two challenging public datasets, demonstrating that GARDEN achieves state-of-the-art performance across both overlap and boundary metrics, with pronounced gains on small lesions and boundary-ambiguous cases.

## 2 Related work

### 2.1 Architectural evolution in plant disease segmentation

Early approaches to plant disease segmentation relied on traditional image processing techniques that were constrained by handcrafted features (Fang et al., 2025; Li et al., 2025), exhibiting poor robustness in complex agricultural environments (Camargo and Smith, 2009; Barbedo, 2013; Lu et al., 2025). The advent of deep learning, particularly the U-Net architecture (Ronneberger et al., 2015) with its seminal encoder-decoder structure and skip connections, established a new benchmark for this task. However, the direct application of standard U-Net to field imagery revealed three principal challenges that catalyzed subsequent research: interference from complex backgrounds, significant variations in the scale of the lesion, and ambiguity at the boundaries of the disease.

To address these challenges, a primary research focus was on enhancing the U-Net architecture. For robustness against complex backgrounds and variable illumination, models began incorporating residual connections (ResBlocks) to build deeper networks (Chen et al., 2022) and attention mechanisms to accentuate salient features (Wang et al., 2024). To tackle drastic scale variation, researchers integrated multi-scale feature extraction modules, such as Atrous Spatial Pyramid Pooling (ASPP) (Wang et al., 2024) and and the Receptive Field Block (RFB) module, which expands the network’s receptive field to achieve multi-scale feature extraction for more effective disease classification (Ding et al., 2023). Recent studies have begun to explore unified frameworks to tackle these challenges simultaneously. For instance, EMSAM (Li et al., 2025) enhances the Segment Anything Model (SAM) (Kirillov et al., 2023) with multi-scale adaptation capabilities, integrate Multi-scale Exponential Moving Average (EMA) (Liu et al., 2025) attention with deformable high-frequency enhancement to jointly address scale variations and edge details. Similarly, KBNet (Yan et al., 2025) employs a language-vision fusion approach to guide segmentation using multimodal cues. However, these methods often rely on heavy pre-trained backbones like SAM or require additional modalities like text prompts. In contrast, we propose a streamlined, purely vision-based architecture that synergistically couples multi-scale pyramid pooling with explicit gradient-guided boundary scanning, ensuring precise segmentation without the computational overhead of heavy transformers or the need for auxiliary modalities.

### 2.2 State space models and Mamba-based segmentation architectures

While Transformers excel at modeling long-range dependencies through self-attention, their quadratic computational complexity poses significant challenges for high-resolution image processing. This limitation has motivated the exploration of State Space Models (SSMs), particularly Mamba, which achieves linear time complexity while maintaining powerful sequence modeling capabilities through selective state spaces and hardware-aware designs.

The adaptation of Mamba to computer vision has gained momentum. Vision Mamba (Zhu et al., 2024b) pioneered the integration of bidirectional selective state space mechanisms with position embeddings, demonstrating that SSMs can serve as effective vision backbones without relying on self-attention. VMamba (Liu et al., 2024b) further refined this approach by introducing cross-scan mechanisms to better capture spatial dependencies in 2D images. These foundational works established that Mamba-based architectures can achieve competitive or superior performance compared to Vision Transformers while maintaining significantly lower computational costs.

Building upon these advances, researchers have developed numerous Mamba-based U-Net variants for semantic segmentation. In the biomedical domain, U-Mamba (Ma et al., 2024) introduced a hybrid CNN-SSM architecture that synergistically combines local feature extraction with global dependency modeling, achieving state-of-the-art performance on multiple medical imaging benchmarks. VM-UNet (Ruan et al., 2024) and SegMamba (Xing et al., 2024) further explored different integration strategies for 3D medical image segmentation, while LoG-VMamba (Dang et al., 2024) addressed the challenge of maintaining both local and global contexts through novel token organization strategies. In agricultural applications, UNetMamba (Zhu et al., 2024a) adapted Mamba for high-resolution remote sensing, and MAVM-UNet (Zhang et al., 2025), CM-UNet (Liu et al., 2024a), and EGCM-UNet (Zheng et al., 2025) integrated multi-scale aggregation and edge guidance for crop disease detection.

Additionally, GMamba (Zhang and Mu, 2024) introduced a state space model combined with convolutions specifically for grape leaf disease segmentation, and LightM-UNet (Liao et al., 2024) explored using Mamba as a lightweight assistant within the U-Net framework.

Despite these advances, a critical gap remains. Most existing Mamba-based methods treat the SSM primarily as a generic context extractor. They lack a targeted mechanism to explicitly condition the selective scan using boundary priors. Without this guidance, the Mamba block may not effectively differentiate between boundary-uncertain pixels and confident interiors. This motivates our framework, which synergistically integrates multi-scale context with a boundary-aware selective scanning mechanism to explicitly refine object contours.

## 3 Methods

This section elaborates on the technical details of our proposed novel deep learning model, Gradient-guided boundary-Aware Region-Driven Edge refinement Network (GARDEN), which is designed to address the challenges of blurred boundaries and scale diversity in plant disease segmentation.

### 3.1 Overall architecture

Initially, the image is fed into a convolutional layer to extract foundational features. It subsequently passes through four downsampling blocks … to sequentially produce hierarchical feature maps, denoted as $f_{e}^{1}$ to $f_{e}^{5}$. The deepest feature map, $f_{e}^{5}$ is then enhanced by the Multi-Scale Context Aggregation (MSCA) to capture rich contextual information across multiple scales.

Following this, the model progressively restores the feature map resolution via four upsampling stages. Within each stage, feature maps from the corresponding encoder level are concatenated along the channel dimension with those from the preceding decoder layer. This fusion is facilitated by a Channel Transformer mechanism, wherein the encoder features are first refined by a Channel-wise Cross-Attention (CCA) module before being combined. The resulting fused feature map is then processed by our core Boundary-aware Selective Scanning (BASS), which leverages boundary detection outcomes to adaptively sharpen lesion contours. Finally, the refined features are passed through a 1 × 1 convolutional layer and a Sigmoid activation function to generate the final segmentation mask of shape [*B*, 3*, H, W*].

The overall workflow of the model is divided into three core parts:

1. Encoder Path: This path is responsible for extracting hierarchical deep features from the input image. It consists of four consecutive DownBlocks. Each DownBlock contains a max-pooling layer with a stride of 2 and two serial convolutional layers (nConvs). Through this process, the spatial resolution of the feature maps is progressively reduced ( H→H/2→…→H/16), while the channel dimension is correspondingly increased, thereby capturing higher-level, more abstract semantic information at the cost of spatial detail. We denote the feature map output by the *i*-th DownBlock as $\;f_{e}^{i}$.
2. Bottleneck: At the deepest level of the encoder path, we introduce the Multi-Scale Context Aggregation (MSCA). This module is designed to process the highest-level semantic features $f_{e}^{5}$ from the encoder, significantly enhancing the model’s adaptability to target scale variations by concatenating contextual information from different scales in parallel along the channel dimension.
3. Decoder Path: This path is dedicated to progressively restoring the abstract features extracted by the encoder to the original image resolution and accurately localizing the lesion boundaries. The decoder is composed of four UpBlocks, symmetric to the encoder. In each upsampling stage, the low-resolution feature map from the previous stage is first upsampled to double its size. Subsequently, to compensate for the spatial details lost during downsampling, feature maps $f_{e}^{i}$ from the corresponding encoder levels are introduced via skip connections. Before fusion, the encoder features are refined by a Channel Attention module to adaptively emphasize more informative channels. The fused feature map is then fed into our core innovative component, the Boundary-aware Selective Scanning (BASS), for refining boundary details, and finally processed through nConvs layers for feature consolidation.

### 3.2 Multi-scale context aggregation

Plant disease spots exhibit significant variations in size and shape across different developmental stages or types. Traditional convolutional networks with fixed receptive fields struggle to efficiently capture both tiny early-stage lesions and large late-stage infections simultaneously. To address this, we design an MSCA module that enables the model to aggregate multi-scale contextual information within a single feature layer.

**Figure 2:**
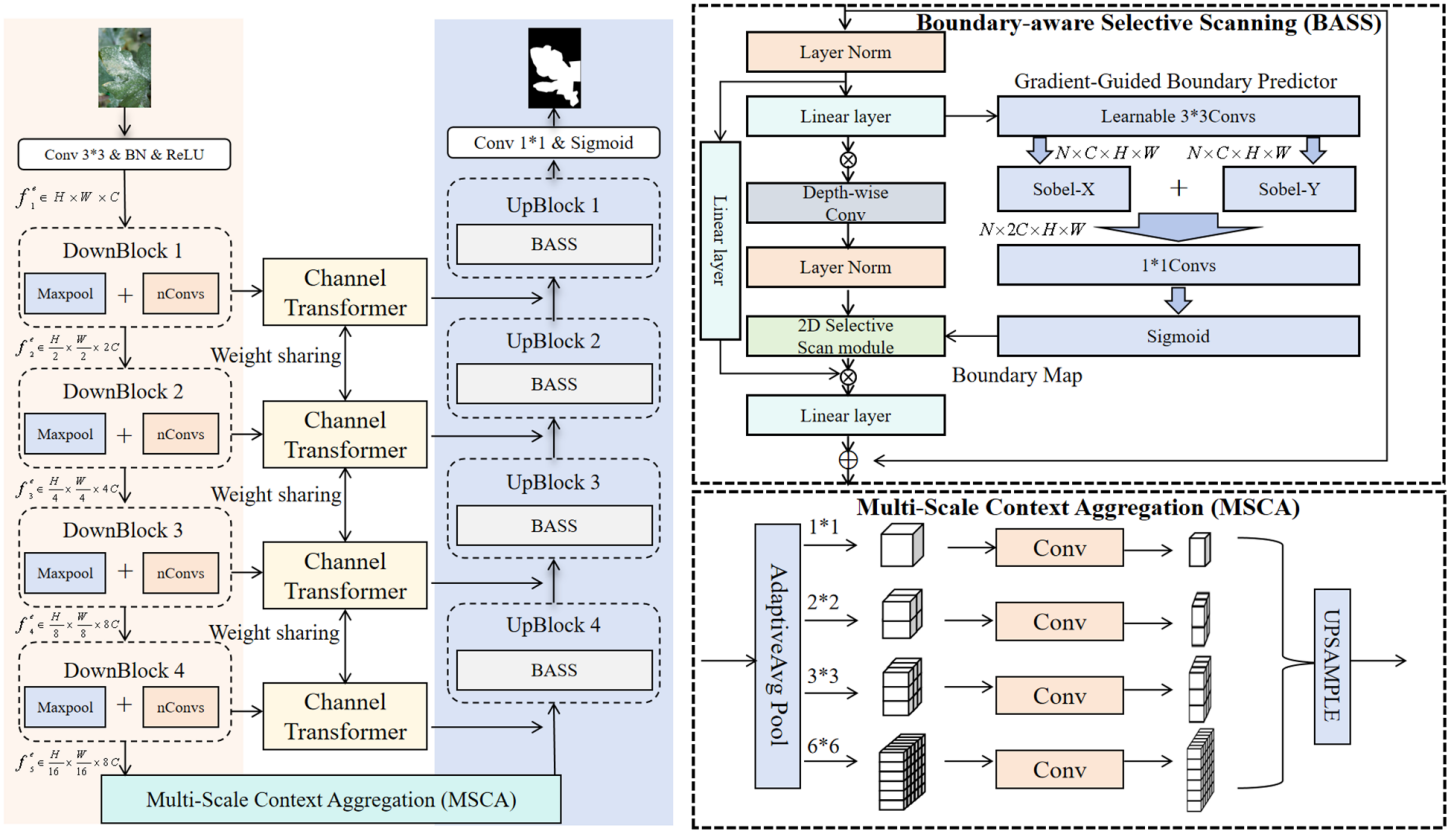
The overall architecture of the proposed GARDEN. The model adopts an encoder-decoder structure. The encoder progressively extracts multi-scale features. The Multi-Scale Context Aggregation (MSCA) (Yu et al., 2018) is applied at the bottleneck to capture rich contextual information. The decoder restores feature resolution, where the core Boundary-aware Selective Scanning (BASS) is utilized to refine boundary details with the guidance of skip connections.

As shown in the bottom-right of **Figure 2**, MSCA receives the feature map $f_{in}\in {\mathbb{R}}^{C\times H^{\prime }\times W^{\prime }}$ from the deepest layer of the encoder. Its core mechanism is a parallel multi-branch pooling structure: specifically, four Adaptive Average Pooling branches compress the input into fixed sizes of 1 × 1, 2 × 2, 3 × 3, and 6 × 6 to capture global, sub-regional, and local context. The output of each branch is then passed through a 1 × 1 convolution to reduce channel dimensions (from *C* to *C/*4) and integrate information. All branch outputs are subsequently upsampled to the original spatial resolution $\left(H^{\prime },W^{\prime }\right)$ via bilinear interpolation and concatenated with the original input feature map *f_in_* along the channel dimension.

The output f_MSCA_ thus fuses rich contextual information from global to local scales. Formally,


$$f_{\text{MSCA}}=\text{Concat }\left(f_{in},\underset{s\in \left\{1,2,3,6\right\}}{⊕}\text{Upsample }\left({\text{Conv}}_{1\times 1}\left({\text{AvgPool}}_{s}\left(f_{in}\right)\right)\right)\right),$$

where AvgPool*_s_* denotes adaptive average pooling with an output size of s×s, ⊕ represents the parallel processing of the branches, and Concat is concatenation along the channel dimension. This design ensures a comprehensive perception of target scale diversity before the features enter the decoder.

### 3.3 Boundary-aware selective scanning with a gradient-guided boundary predictor

Decoder upsampling and feature fusion tend to blur object boundaries, which undermines precise quantification of lesion area and morphology. We introduce BASS, a plug-and-play refinement module that (i) explicitly predicts a boundary prior and (ii) conditions a long-range 2D Mamba selective scan on this prior to sharpen contours during feature reconstruction. BASS comprises two components: a Gradient-Guided Boundary Predictor (GGBP) and a Boundary-Conditioned 2D Mamba Selective Scan.

#### 3.3.1 Gradient-guided boundary predictor

Given a stage feature $X_{\text{in}}\in {\mathbb{R}}^{C\times H\times W}$, GGBP produces a continuous boundary prior $M_{b}\in {\left[0,1\right]}^{1\times H\times W}$. As illustrated in the top-right of **Figure 2**, we apply two parallel learnable  3X3 convolutions initialized as Sobel- x and Sobel- y operators to approximate horizontal and vertical gradient responses:


$$G_{x}\;=\;{ℱ}_{x}\left(X_{\text{in}}\right),\;G_{y}\;=\;{ℱ}_{y}\left(X_{\text{in}}\right).$$

The two responses are concatenated and fused by a 1×1 convolution, followed by a sigmoid to obtain a single-channel boundary prior:


$$M_{b}\;=\;\sigma \;\left({\text{Conv}}_{1\times 1}\;\left(\text{Concat}\left(G_{x},\;G_{y}\right)\right)\right).$$

This design preserves continuous gradient magnitude/orientation cues rather than collapsing them into a binary edge mask, providing richer and more stable guidance for the subsequent selective scanning. The Sobel initialization injects an edge-aware inductive bias at the start of training while allowing the filters to adapt to task-specific boundary patterns end-to-end.

#### 3.3.2 Boundary-conditioned 2D Mamba selective scan

Let $\Phi_{\text{M}}^{\left(d\right)}\left(\cdot \right)$ denote the 2D Mamba selective-scan operator applied along direction d. We first project $X_{\text{in}}$ to the model dimension via a 1×1 convolution, $\hat{X}=\text{Proj}\left(X_{\text{in}}\right)$, and perform four directional scans to mitigate directional bias:


$$Z^{\left(d\right)}\;=\;\Phi_{\text{M}}^{\left(d\right)}\left(\hat{X}\right),\;d\in \left\{↗,↘,↖,↙\right\}$$

To inject boundary awareness, we convert *M_b_* into a gating field with learnable affine parameters  (γ,β):


$$\alpha \;=\;\sigma \;\left(\gamma \;M_{b}+\beta \right),\;\alpha \in {\left[0,1\right]}^{1\times H\times W}$$

and blend long-range scanned features with local evidence in a boundary-conditioned residual form:


$${\tilde{Z}}^{\left(d\right)}\;=\;\left(1-\alpha \right)\;⊙\;\hat{X}\;+\;\alpha \;⊙\;Z^{\left(d\right)}.$$

Finally, the four directional results are fused and projected back to C channels, followed by a residual connection to preserve semantics:


$$X_{\text{out}}\;=\;X_{\text{in}}\;+\;{\text{Proj}}^{-1}\;(\text{Fuse}({\left\{{\tilde{Z}}^{\left(d\right)}\right\}}_{d})).$$

Intuitively, pixels with high boundary likelihood (large *α*) receive stronger long-range support to resolve contour ambiguity, whereas confident interiors rely primarily on local cues, improving both efficiency and edge fidelity. Applied at successive decoder stages, BASS consistently enforces boundary precision while complementing multi-scale context cues provided by MSCA.

## 4 Experiment

This section systematically evaluates the performance of our proposed model (hereafter referred to as Ours). We first introduce the datasets and evaluation metrics, followed by the implementation details. All experiments were conducted using the PyTorch deep learning framework on a workstation equipped with an NVIDIA RTX 2080 Ti GPU with 22GB of VRAM. During the training process, all input images were resized to a uniform resolution of 224 × 224 pixels. We employed the Adam optimizer with an initial learning rate of 1 × 10^−4^ and utilized a ReduceLROnPlateau learning rate scheduler to stabilize training, which reduces the learning rate by a factor of 0.5 if the validation loss does not improve for a patience of 10 epochs. The loss function was a weighted sum of DiceLossWithLogits and CrossEntropyLoss, with a weight of 0.5 for each, to simultaneously optimize for pixel-level classification accuracy and region-level segmentation consistency. The model was trained for 100 epochs with a batch size of 8, and data preprocessing strategies included resizing, conversion to tensors, and normalization to the range. Subsequently, we present a quantitative comparison of our model against several state-of-the-art segmentation models. Finally, through a series of comprehensive ablation studies, we validate the effectiveness of each innovative component within our model and provide an in-depth discussion of its behavior and advantages, supported by qualitative visualizations.

### 4.1 Dataset

To comprehensively evaluate the effectiveness and generalization capability of our proposed model, we utilized two publicly available plant disease segmentation datasets, each presenting distinct challenges.

#### 4.1.1 Leaf disease segmentation dataset

The Leaf Disease Segmentation Dataset is a benchmark collection widely utilized for research in plant lesion segmentation. It comprises a total of 2,940 images of diseased leaves captured against complex backgrounds, featuring lesions with heterogeneous shapes, sizes, and colors (Lu et al., 2023). The dataset is officially divided into a training set of 1,764 images, a validation set of 588 images, and a test set of 588 images. For our experiments, we adhere to these official splits provided by the dataset. Each image is accompanied by a corresponding pixel-level annotation mask. The primary challenges presented by this dataset are the often indistinct boundaries between lesions and healthy plant tissue, as well as significant color variations resulting from natural lighting conditions. Notably, the images in this dataset are not of fixed resolution, varying from approximately 256 × 256 to 1200 × 1009 pixels. This variability necessitates a model capable of handling diverse spatial scales.

**Figure 3:**
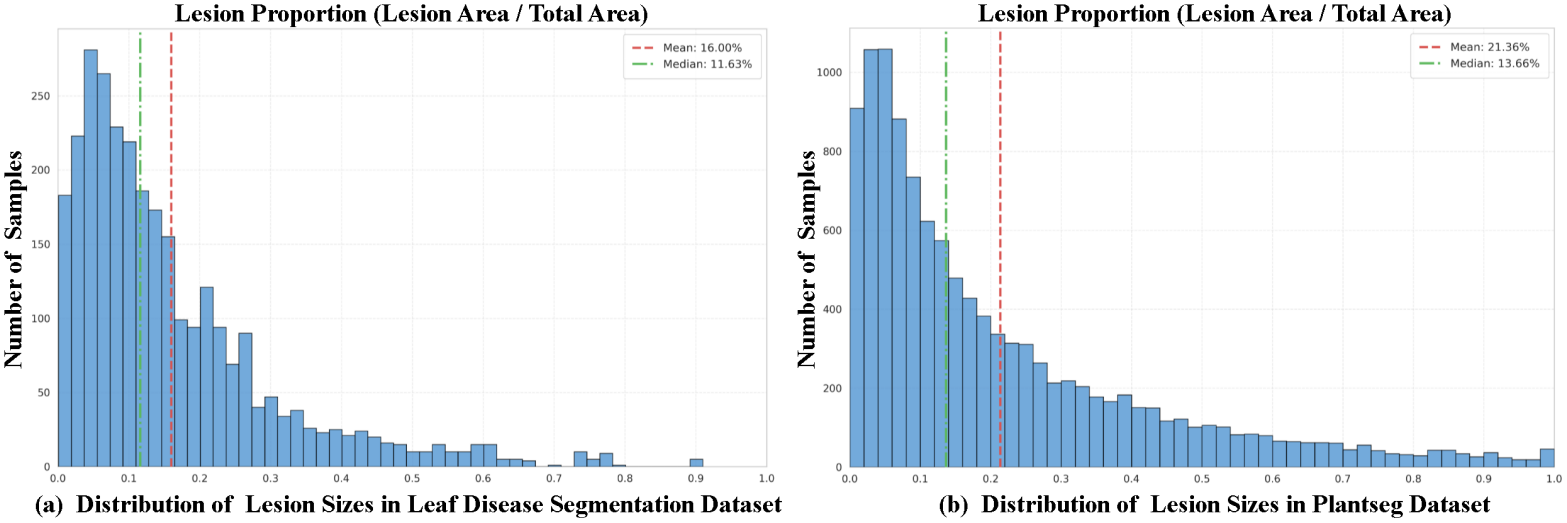
Quantitative analysis of lesion scale distribution. **(A)** The Leaf Disease Segmentation Dataset and **(B)** the PlantSeg Dataset. The histograms illustrate the frequency distribution of lesion proportions, calculated as the ratio of lesion pixels to the total image resolution for each sample. The red dashed line and green dash-dot line represent the mean and median proportions, respectively. The high Standard Deviation (Std Dev) and the wide range quantitatively confirm the drastic scale variation and the long-tail distribution inherent in both datasets.

To strictly quantify the challenge of scale variation, we calculated the pixel-wise lesion proportion for every image in the dataset. As visualized in (**Figure 3a**), the analysis reveals a standard deviation of 14.62%, indicating substantial variability in lesion sizes across the dataset. Furthermore, the discrepancy between the mean (16.00%) and median (11.63%) indicates a pronounced right-skewed distribution, confirming that the dataset contains a complex mix of numerous minute spots and large coalesced necrotic regions.

#### 4.1.2 PlantSeg dataset

The PlantSeg (Wei et al., 2024) dataset, a large-scale collection of images captured “in-the-wild,” poses a greater challenge to model robustness and generalization. In contrast to datasets gathered under controlled laboratory settings, PlantSeg features more complex backgrounds (e.g., soil, weeds, other plant organs) and highly variable illumination (e.g., shadows, highlights). Furthermore, the disease lesions are frequently occluded or appear in scattered, non-contiguous patterns. The dataset is comprehensive and is partitioned into 7,916 images for training, 1,247 for validation, and 2,295 for testing, encompassing 115 distinct types of plant diseases. We utilize the official partitions provided by the dataset in our work. Therefore, evaluation on PlantSeg serves as a rigorous test of a model’s performance in scenarios that closely mimic real-world agricultural applications. The dataset also exhibits significant diversity in image resolution, with dimensions typically ranging from 256 × 256 to 1200 × 1009 pixels, reflecting real-world inconsistencies in data acquisition.

Similarly, the quantitative analysis presented in (**Figure 3b**) demonstrates even more extreme heterogeneity. The PlantSeg dataset exhibits a higher standard deviation of 21.30%. Moreover, the significant divergence between the mean (21.36%) and the median (13.66%) highlights a heavily skewed distribution with frequent occurrences of both minute and extensive lesions. This implies that the model must be capable of segmenting targets across a vast spectrum of scales, providing strong empirical justification for our multi-scale architectural design.

### 4.2 Evaluation metrics

To quantitatively assess the segmentation performance, we employed five commonly used metrics in the field of image segmentation: the Dice Coefficient (Dice), Mean Intersection over Union (mIoU), Precision, and Recall. Among these, the Dice Coefficient and mIoU serve as the primary indicators of the model’s overall performance.

Dice Coefficient (Dice): The core metric for measuring the overlap between the predicted segmentation (*P*) and the ground truth (*G*). It ranges from 0 to 1, where a higher value indicates better segmentation.


$$\text{Dice}=\frac{2\times \left|P\cap G\right|}{\left|P\right|+\left|G\right|}=\frac{2\times \text{TP}}{2\times \text{TP}+\text{FP}+\text{FN}}$$

Mean Intersection over Union (mIoU): Another key metric that calculates the ratio of the intersection to the union of the predicted and ground truth regions.


$$\text{mIoU}=\frac{\left|P\cap G\right|}{\left|P\cup G\right|}=\frac{\text{TP}}{\text{TP}+\text{FP}+\text{FN}}$$

Precision: Measures the proportion of correctly identified positive pixels among all pixels predicted as positive. High precision indicates a low false positive rate.


$$\text{Precision}=\frac{\text{TP}}{\text{TP}+\text{FP}}$$

Recall: Measures the proportion of correctly identified positive pixels among all actual positive pixels. High recall indicates a low false negative rate.


$$\text{Recall}=\frac{\text{TP}}{\text{TP}+\text{FN}}$$

Specificity: Measures the proportion of correctly identified negative pixels (background) among all actual negative pixels.


$$\text{Specificity}=\frac{\text{TN}}{\text{TN}+\text{FP}}$$

where TP, FP, and FN represent the number of true positive, false positive, and false negative pixels, respectively.

### 4.3 Comparison experiments

To comprehensively validate the effectiveness and superiority of our proposed model (hereafter referred to as Ours), we conducted extensive quantitative comparisons against several mainstream semantic segmentation models on two datasets with distinct characteristics. The compared models include classical architectures like U-Net, attention-based models such as Att-UNet (Oktay et al., 2018), U-Net++ (Zhou et al., 2019), and MultiResUNet (Ibtehaz and Rahman, 2020), as well as Transformer-based models including TransUNet (Chen et al., 2021), UCTransNet (Wang et al., 2022), MISSFormer (Huang et al., 2021), DA-TransUNet (Sun et al., 2024), and Mamba-based model including EBMA-UNet (Shu et al., 2025), LightM-UNet (Liao et al., 2024).

Among these, LightM-UNet represents a cutting-edge Mamba-based architecture designed for medical image segmentation. While it introduces the efficiency of State Space Models, our experiments indicate that its lightweight design struggles to capture the ambiguous boundaries of plant lesions compared to our method, as it lacks a specific boundary-guided mechanism.

The quantitative results are presented in **Tables 1**, **2**. First, on the widely-used Leaf Disease benchmark dataset (as shown in **Table 1**), our model demonstrates state-of-the-art (SOTA) performance across all evaluation metrics. Specifically, on the core segmentation metrics of Dice coefficient and mIoU, our model achieves scores of 88.84% and 87.99%, respectively. This represents a substantial improvement of 8.37 percentage points in Dice score over the classic U-Net baseline (80.47%). Furthermore, even when compared to the strong UCTransNet model (Dice 87.34%), our model maintains a solid advantage, proving the superiority of our architectural design.

**Table 1:** Experimental results on the leaf disease segmentation dataset.

Model	Accuracy	Dice	Precision	Specificity	Recall	mIoU
U-Net	93.90	80.47	81.15	96.58	79.80	80.22
Att-UNet	95.30	84.43	87.29	97.80	81.75	83.84
TransUNet	95.18	84.42	85.06	97.28	83.79	83.75
UCTransNet	96.09	87.34	88.07	97.83	86.63	86.51
MultiResUNet	91.96	76.52	70.17	93.40	84.13	76.36
U-Net++	94.77	82.93	84.11	97.22	81.49	82.43
MISSFormer	92.40	75.07	76.77	95.90	73.44	75.76
DA-TransUNet	92.71	73.84	83.66	97.62	66.09	75.20
EBMA-UNet	90.84	68.27	74.14	95.93	63.27	70.84
LightM-UNet	87.54	57.48	61.32	93.70	54.10	63.36
Ours	96.58	88.84	90.44	98.30	87.29	87.99

Bold values indicate the best performance in each column.

**Table 2:** Experimental results on the PlantSeg dataset.

Model	Accuracy	Dice	Precision	Specificity	Recall	mIoU
U-Net	86.64	67.50	69.14	92.16	65.94	67.72
Att-UNet	88.09	71.76	71.60	92.40	71.92	70.96
TransUNet	89.03	72.88	73.30	93.26	**72**.**46**	72.23
UCTransNet	88.95	72.30	73.73	**95**.**54**	70.93	71.85
MultiResUNet	85.65	65.96	65.86	90.87	66.07	66.27
U-Net++	87.47	69.61	71.07	92.60	68.21	69.38
MISSFormer	81.75	53.43	57.70	90.28	49.76	58.04
DA-TransUNet	86.09	66.19	67.74	91.79	64.70	66.68
EBMA-UNet	86.85	66.90	69.01	92.22	65.03	67.35
LightM-UNet	85.17	62.16	67.14	92.45	57.86	64.11
Ours	**89.26**	**73.67**	**76.06**	94.01	71.43	**72.84**

Bold values indicate the best performance.

Subsequently, to further test the model’s robustness and generalization capability in complex, real-world scenarios, we conducted evaluations on the more challenging “in-the-wild” PlantSeg dataset (as shown in **Table 2**). The complex backgrounds and variable lighting conditions of this dataset posed a greater challenge for all models, leading to a general decrease in scores across all metrics compared to the Leaf Disease dataset. However, even under these stringent conditions, our model maintained its leading position, achieving the highest Dice score of 73.67% and an mIoU of 72.84%, once again surpassing all compared models. This result strongly demonstrates that our model not only excels on benchmark datasets but also possesses strong stability and reliability in environments that simulate real-world applications.

**Figure 4:**
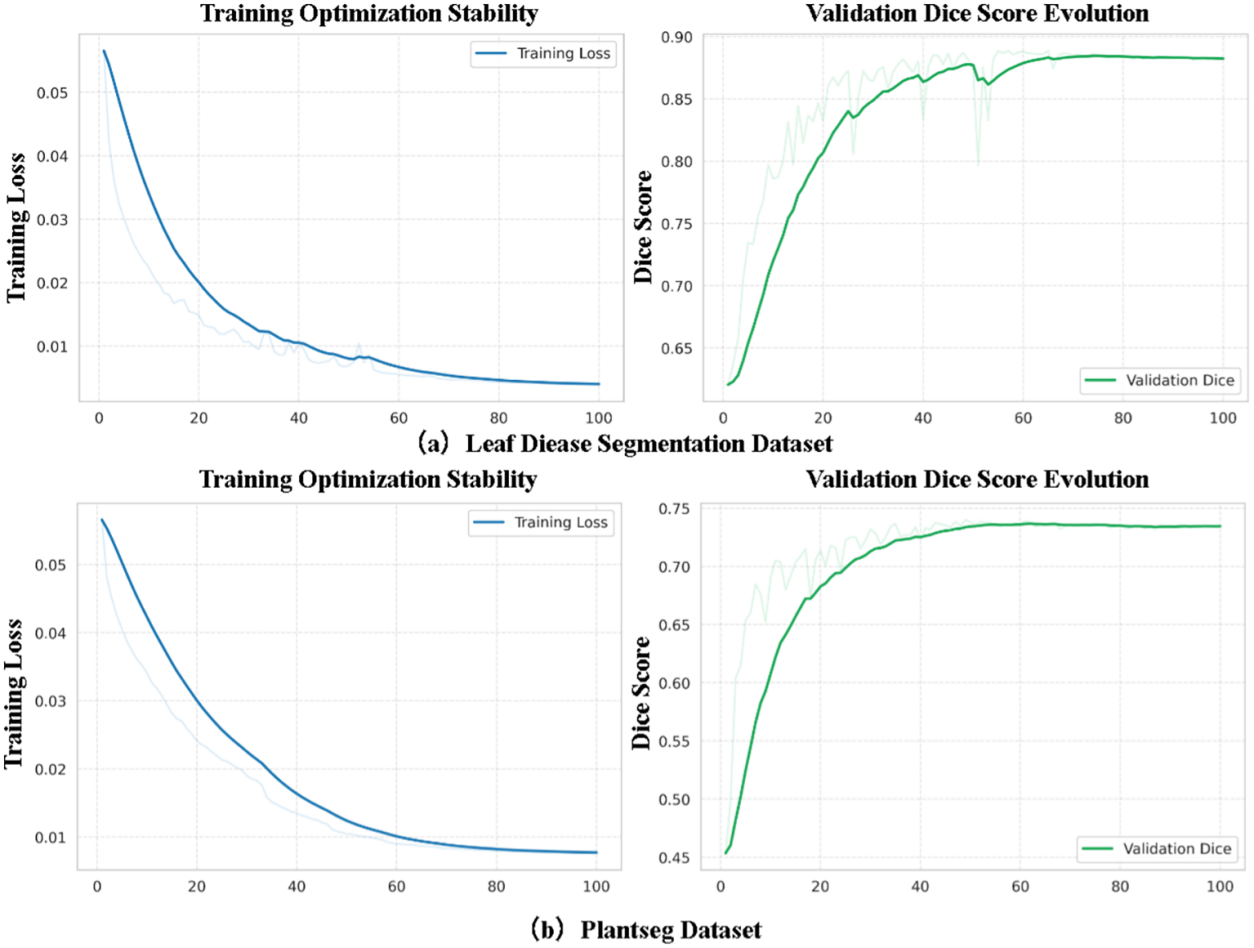
Visualization of training convergence and stability analysis. **(A)** Training dynamics on the Leaf Disease Segmentation Dataset. **(B)** Training dynamics on the PlantSeg Dataset. The smooth descent of the training loss (left column) and the consistent improvement of the validation Dice score (right column) demonstrate the optimization stability of the proposed GARDEN model.

Beyond static performance comparisons, we further investigated the training dynamics to verify the optimization stability of our Mamba-based architecture, addressing potential concerns regarding the convergence challenges of State Space Models. As illustrated in **Figure 4**, the training loss for both the Leaf Disease dataset (**Figure 4a**) and the PlantSeg dataset (**Figure 4b**) exhibits a smooth, monotonic descent without significant oscillations or divergences. Concurrently, the validation Dice scores steadily improve and converge to their optimal values. These empirical results confirm that the introduction of the selective scanning mechanism (BASS) maintains robust numerical stability and does not disrupt the gradient flow during optimization.

In summary, the quantitative comparison experiments on both datasets consistently demonstrate that our proposed model is significantly superior to existing mainstream methods for plant disease segmentation tasks, in terms of both accuracy in standard scenarios and robustness in complex environments.

### 4.4 Ablation studies

To systematically validate the individual effectiveness and synergistic interplay of our model’s innovative components, and to determine the optimal settings for their key hyperparameters, we conducted a series of comprehensive ablation studies.

#### 4.4.1 Effectiveness of core components

We first assessed the effectiveness of our core components through a component-level ablation study, with the results presented in **Table 3**. Our baseline model is a U-Net variant integrated with the Channel Transformer (CTrans). The introduction of the MSCA module alone yields a stable performance improvement on both datasets (e.g., Dice score increases to 87.56% on Leaf Disease), confirming the importance of multi-scale context at the bottleneck. Interestingly, integrating the BASS module alone resulted in a slight performance degradation. We hypothesize that BASS, as a specialized module for fine-grained boundary refinement, is highly dependent on high-quality deep semantic features. Without the multi-scale context provided by MSCA, BASS might over-optimize on less representative features, leading to suboptimal results. Crucially, when both MSCA and BASS are integrated, the model achieves the best performance by a significant margin (e.g., 88.82% Dice on Leaf Disease). This strongly demonstrates a powerful synergistic effect between our two main innovations: MSCA provides a robust, multi-scale feature foundation, upon which BASS can then perform precise boundary optimization.

**Table 3:** Component-level ablation study.

Dataset	MSCA	BASS	Dice	mIoU
			87.34	86.51
Leaf disease	✓		87.56	86.75
		✓	86.35	85.60
	✓	✓	**88.82**	**87.94**
			72.30	71.85
Plantseg	✓		72.53	71.73
		✓	70.88	70.71
	✓	✓	**73.16**	**72.36**

Bold values indicate the best performance.

#### 4.4.2 Analysis of internal module parameters

Having validated the synergistic effectiveness of our core components, we further conducted fine-grained experiments to investigate the optimal hyperparameter settings for each module, as detailed in **Table 4**, **5**, and **6**.

**Table 4:** Ablation study on different settings of the MSCA module. The best result in each column is highlighted in bold.

Setting	Leaf disease				Plantseg			
	Dice (%)	mIoU (%)	Prec. (%)	Recall (%)	Dice (%)	mIoU (%)	Prec. (%)	Recall (%)
[1] (Global)	87.31	86.46	87.55	87.09	71.05	70.43	71.43	70.67
[1, 2, 6]	**88.84**	**87.99**	**90.44**	**87.29**	72.51	71.89	75.23	69.99
[1, 2, 3, 6]	88.07	87.24	90.06	86.17	**73.67**	**72.84**	76.06	**71.43**
[1, 3, 5]	88.35	87.51	90.09	86.69	71.63	71.34	**76.17**	67.60

Bold values indicate the best performance.

**Table 5:** Ablation study on the kernel size of the boundary predictor.

Kernel size	Leaf disease				Plantseg			
	Dice (%)	mIoU (%)	Prec. (%)	Recall (%)	Dice (%)	mIoU (%)	Prec. (%)	Recall (%)
(3x3)	**88.84**	**87.99**	**90.44**	**87.29**	73.15	72.09	73.15	**72.97**
(5x5)	87.90	87.07	89.59	86.27	**73.67**	**72.84**	**76.06**	71.43

Bold values indicate the best performance.

**Table 6:** Ablation study on the sharpening factor of the boundary modulation.

Sharpening factor	Leaf disease				Plantseg			
	Dice (%)	mIoU (%)	Prec. (%)	Recall (%)	Dice (%)	mIoU (%)	Prec. (%)	Recall (%)
1	**88.84**	**87.99**	**90.44**	87.29	**73.67**	**72.84**	**76.06**	71.43
5	88.82	87.94	89.58	**88.07**	73.15	72.09	73.15	**72.97**
10	88.35	87.50	89.64	87.11	72.66	72.02	75.45	70.06

Bold values indicate the best performance.

For the MSCA module (**Table 4**), the results indicate that a richer pyramid structure, such as [1, 2, 6] or [1, 2, 3, 6], consistently outperforms a single global context, underscoring the necessity of a diverse multi-scale feature representation.

The study on the Boundary Predictor’s kernel size (**Table 5**) revealed a compelling dataset-dependent phenomenon. On the Leaf Disease dataset, a smaller 3 × 3 kernel achieved superior performance, likely due to its focus on local, fine-grained edge details. Conversely, on the more complex “in-the-wild” PlantSeg dataset, a larger 5 × 5 kernel with its wider receptive field proved more robust, suggesting its ability to integrate more context is advantageous for identifying boundaries in noisy environments.

Finally, for the Boundary Modulation (**Table 6**), we investigated the sharpening factor (corresponding to the parameter *γ* in Eq. 4). Founding that an excessively high sharpening factor (e.g., 10) was detrimental to performance. The optimal factor was again dataset-dependent: a moderate factor of 5 was best for the Leaf Disease dataset, while a more conservative factor of 1 was optimal for the noisier PlantSeg dataset. This suggests that a gentler modulation is more robust in complex scenarios to avoid amplifying noise.

### 4.5 Case studies

**Figure 5:**
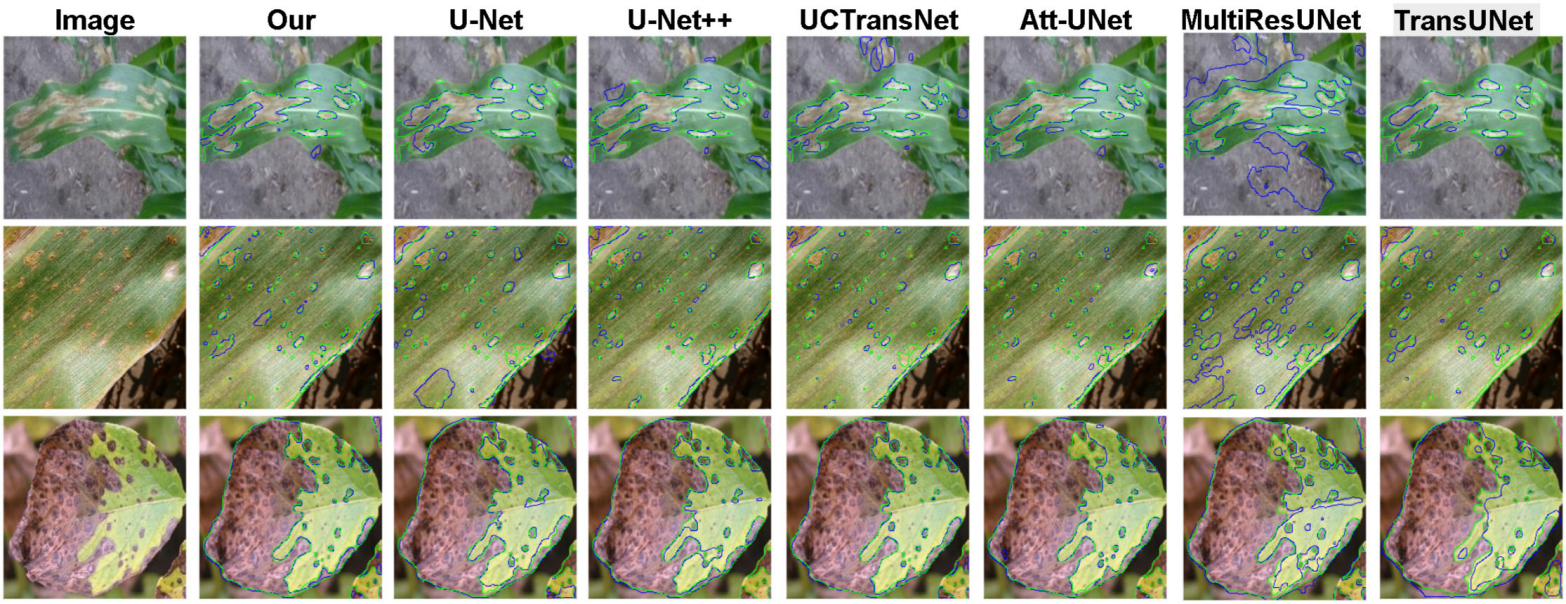
Our model is qualitatively compared with six other mainstream segmentation models on the leaf disease dataset. In the figure, the green line represents the ground truth, and the blue line represents the model predictions.

**Figure 6:**
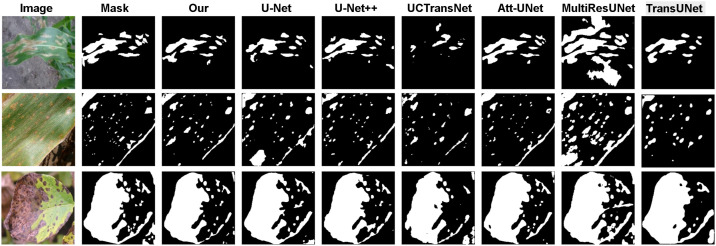
Comparison of binarized masks of segmentation results by different models on the leaf disease dataset.

**Figures 5** through 8 intuitively reveal the superiority of our model in addressing key challenges in plant disease segmentation. In the Leaf Disease dataset (**Figures 5**, **6**), existing methods commonly struggle with the issue of intra-class texture heterogeneity arising from varied disease progression. For instance, U-Net and its variants often lose critical pathological features when confronting large-scale, irregular lesions, resulting in incomplete segmentation masks. In stark contrast, our model leverages an advanced feature fusion mechanism to maintain a high degree of connectivity and internal consistency within the segmented regions, ensuring precise coverage of the entire infected area. Concurrently, by performing fine-grained boundary modeling, our model effectively overcomes the ambiguity at the transition zones between pathogenic and healthy tissues, thereby achieving pixel-level delineation accuracy.

**Figure 7:**
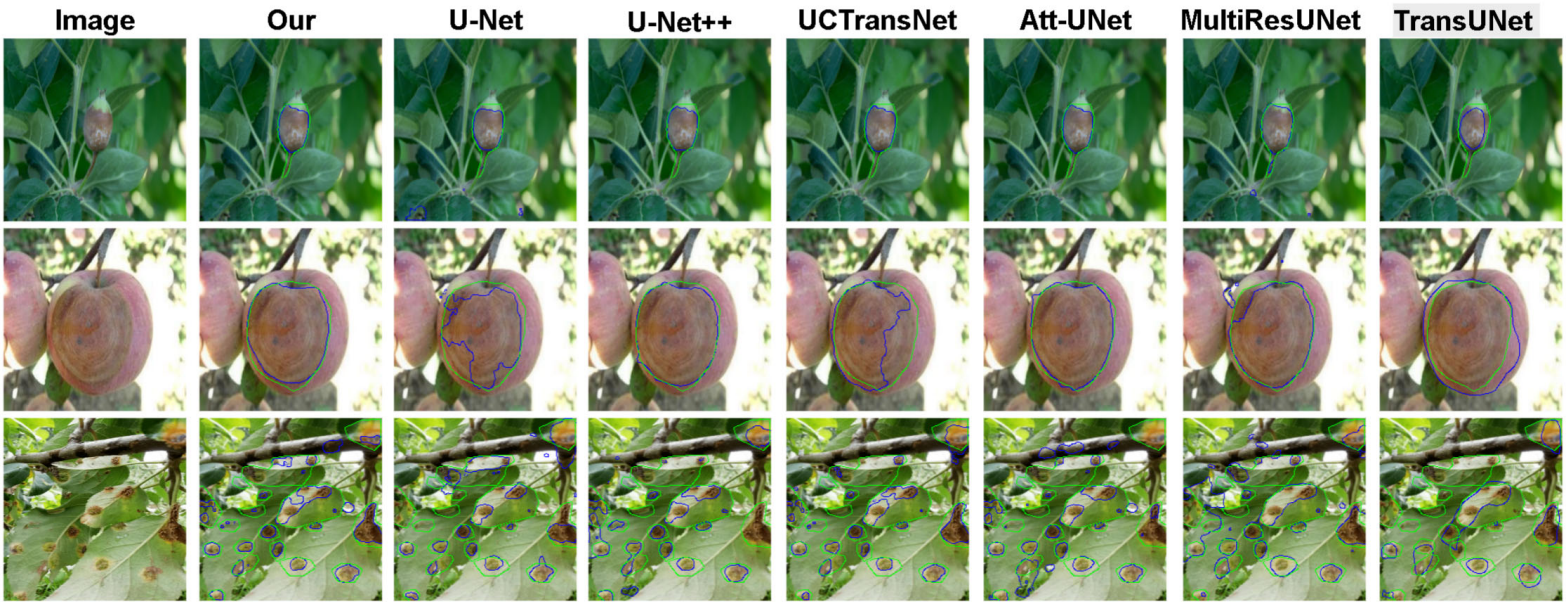
Our model is qualitatively compared with six other mainstream segmentation models on the Plantseg dataset. In the figure, the green line represents the ground truth, and the blue line represents the model predictions.

**Figure 8:**
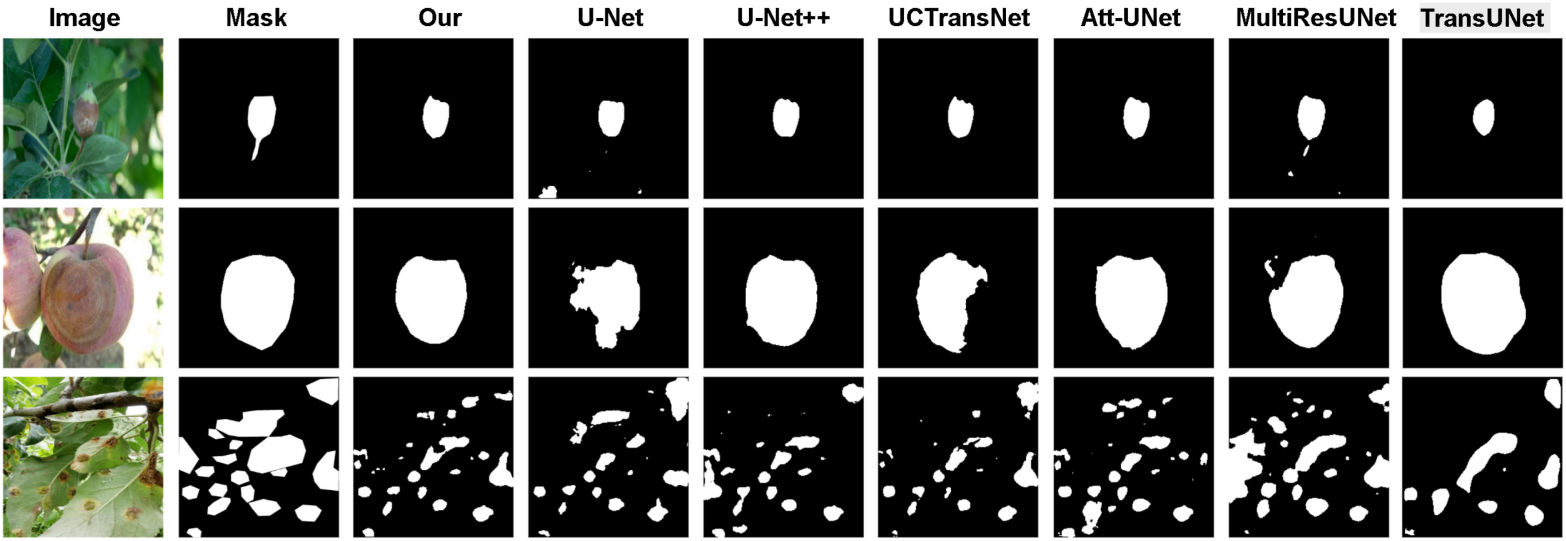
Comparison of binarized masks of segmentation results by different models on the Plantseg dataset.

On the more complex PlantSeg dataset (**Figures 7**, **8**), our model further demonstrates its capability in handling extreme scenarios. A core challenge lies in the detection of diminutive targets, which are highly susceptible to information loss during the down-sampling stages of a network. Our model excels at this task (list 3 of **Figure 8**), where its multi-scale feature learning capability ensures a keen perception of minute lesions. Furthermore, when faced with image degradation factors such as illumination variance, background interference, and low signal-to-noise ratios, our model consistently yields results that are highly congruent with the ground truth, whereas other methods exhibit varying degrees of performance decay. Collectively, this qualitative comparison convincingly demonstrates that our model establishes a new benchmark in segmentation precision, robustness, and generalization capability.

### 4.6 Qualitative visualization

**Figure 9:**
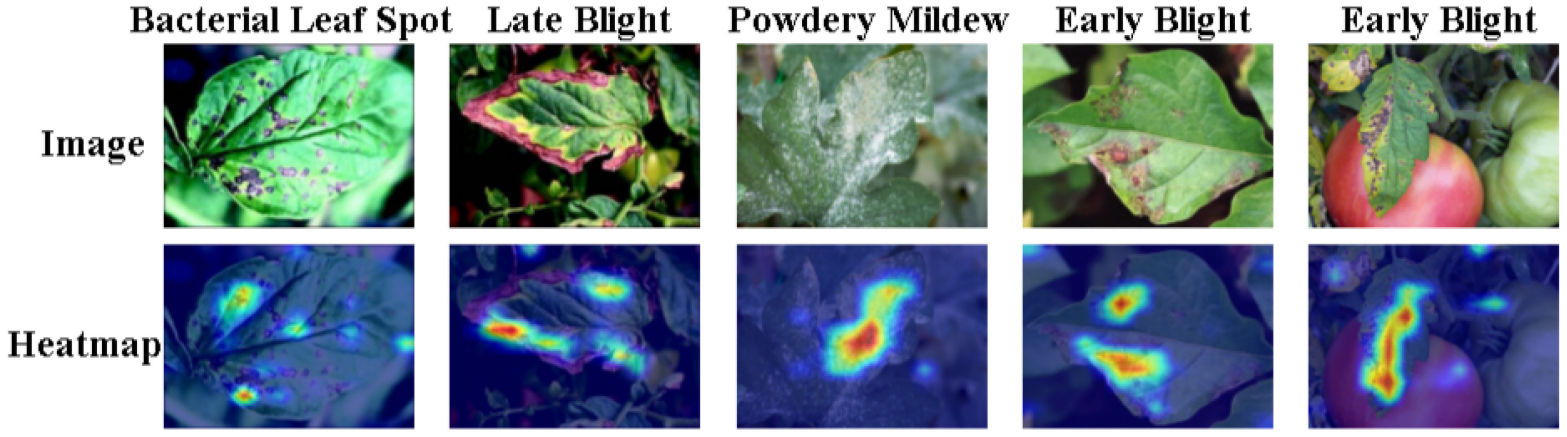
Use Grad-CAM visualization to perform interpretability analysis and compare with actual lesion results.

To further probe the decision-making process of our model, we visualized its regions of focus using Grad-CAM, as depicted in **Figure 9**. The visualizations clearly demonstrate a strong spatial correspondence between the model’s learned features and the ground-truth lesion regions, with activation intensity (red areas) highly concentrated on disease-affected pixels rather than on healthy tissue or the background.

This precise localization capability is validated across a variety of challenging scenarios. For instance, the model accurately focuses its attention on the core lesion areas for both the diffuse small spots of Bacterial Leaf Spot (first column) and the large-area marginal necrosis of Late Blight (second column). Particularly noteworthy is the model’s performance in cases with complex backgrounds or where the lesion color closely resembles that of the leaf—such as in the fifth column where a lesion appears on a tomato fruit, or in the third column featuring Powdery Mildew. In these instances, the model can still effectively ignore irrelevant background information (e.g., the fruit, other healthy leaves) and concentrate its computational resources on identifying pathological features.

This targeted attention mechanism qualitatively validates our model’s robust ability to discriminate key pathological features from confounding factors, which is crucial for its capacity to filter substantial redundant information and ultimately achieve precise segmentation.

### 4.7 Discussion and limitations

This study validates that coupling multi-scale context with boundary refinement sets a new SOTA in plant lesion segmentation. Ablation results highlight the synergy between MSCA and BASS: MSCA provides a robust semantic foundation, which allows BASS to effectively sharpen contours without amplifying noise. Mechanistically, the GGBP-conditioned Mamba scan allocates long-range reasoning specifically to ambiguous boundaries, proving more efficient than global attention. Ultimately, GARDEN demonstrates that a “semantics-first, refinement-second” approach is essential for handling the extreme scale variations and fuzzy boundaries inherent in plant pathology.

Despite these strengths, we identify certain limitations and observations that merit discussion. First, regarding model interpretability, we observed minor activation artifacts in non-lesion regions within the Grad-CAM visualizations **Figure 9**. We attribute this to the high sensitivity of the BASS module’s selective scanning mechanism combined with MSCA’s global aggregation. The model aggressively evaluates all regions with textures resembling lesions to ensure no subtle disease features are missed. Furthermore, the significant variance in original image resolutions necessitates resizing to a uniform input dimension during training; this resampling process may introduce aliasing or interpolation noise that manifests as low-level background activations. While this generates low-confidence background noise in the heatmap, it fundamentally reflects the model’s strong capability in capturing global context and boundary details. Crucially, the internal gating mechanism effectively filters these signals, ensuring they do not result in false positives in the final segmentation mask.

Second, despite Mamba’s linear complexity, the current GARDEN architecture requires optimization for strict edge-computing scenarios. Future work will explore model compression techniques to further reduce computational costs. Finally, the model exhibits sensitivity to hyperparameters across different domains (e.g., varying kernel sizes for lab vs. field data). While adaptable, this currently requires manual tuning. We plan to investigate adaptive mechanisms to automate this calibration for diverse agricultural environments.

## 5 Conclusion

In this work we addressed two persistent challenges in plant lesion segmentation—drastic variation in lesion scale and ambiguous, low-contrast boundaries—by introducing GARDEN, a gradient-guided, boundary-aware, region-driven edge-refinement network. At its core, the Multi-Scale Context Aggregation (MSCA) module gathers contextual cues over diverse receptive fields to form scale-consistent lesion priors, enabling robust detection from minute early spots to large coalesced regions. Complementing this, the Boundary-aware Selective Scanning (BASS) module, conditioned on a Gradient-Guided Boundary Predictor (GGBP), allocates long-range reasoning selectively to boundary-uncertain pixels while relying on local evidence in confident interiors. This coupling of cross-scale reasoning with boundary-aware refinement yields precise masks and sharper contours across challenging imaging conditions.

Comprehensive experiments on two public datasets show that GARDEN achieves state-of-the-art performance on both overlap- and boundary-sensitive metrics, with particularly strong gains on small lesions and cases with weak or fuzzy edges. Qualitative analyses further indicate that the model preserves fine structures without sacrificing the integrity of large lesions. The design is modular and easily integrated into standard encoder–decoder frameworks, providing a practical recipe for improving plant disease segmentation systems used in precision agriculture.

GARDEN currently derives an explicit boundary prior from image gradients; while effective, this cue can be sensitive to illumination changes and annotation noise. Future work will investigate learned or multi-cue boundary supervision, uncertainty estimation to flag ambiguous regions, and active learning strategies that prioritize boundary annotations. Extending the approach to cross-domain settings (different species, cultivars, and sensors) via domain adaptation or test-time training is another promising direction. Finally, exploring efficiency–accuracy trade-offs for on-device deployment and incorporating additional modalities (e.g., hyperspectral or thermal cues) may further enhance robustness in real-world field conditions.

## Data Availability

Publicly available datasets were analyzed in this study. This data can be found here: https://www.kaggle.com/datasets/fakhrealam9537/leaf-disease-segmentation-datasethttps://zenodo.org/records/13762907.
